# Extremely rare coronary anomaly as cause of palpitations: a case report of single right coronary artery—multimodality imaging and its impact on treatment decisions

**DOI:** 10.1093/ehjcr/ytae584

**Published:** 2024-10-28

**Authors:** Alexander Suchodolski, Natasza Millan, Katarzyna Mykieta, Jan Głowacki, Mariola Szulik

**Affiliations:** Doctoral School of the Medical University of Silesia in Katowice, ul. Poniatowskiego 15, 40-055 Katowice, Poland; Department of Cardiology and Electrotherapy, Faculty of Medical Sciences in Zabrze, Silesian Center for Heart Diseases, Medical University of Silesia, ul. Marii Curie Skłodowskiej 9, 41-800 Zabrze, Poland; Student Research Group, Faculty of Medical Sciences in Zabrze, Medical University of Silesia, ul. Marii Curie Skłodowskiej 9, 14-800 Zabrze, Poland; Student Research Group, Faculty of Medical Sciences in Zabrze, Medical University of Silesia, ul. Marii Curie Skłodowskiej 9, 14-800 Zabrze, Poland; Department of Radiology and Radiodiagnostics, Faculty of Medical Sciences in Zabrze, Medical University of Silesia, ul. Marii Curie Skłodowskiej 9, 41-800 Zabrze, Poland; Computed Tomography Laboratory, Silesian Centre for Heart Diseases, ul. Marii Curie Skłodowskiej 9, 41-800 Zabrze, Poland; Department of Cardiology and Electrotherapy, Faculty of Medical Sciences in Zabrze, Silesian Center for Heart Diseases, Medical University of Silesia, ul. Marii Curie Skłodowskiej 9, 41-800 Zabrze, Poland; Department of Medical and Health Sciences, WSB University Faculty of Applied Sciences, ul. Cieplaka 1C, 41-300 Dąbrowa Górnicza, Poland

**Keywords:** Case report, Single right coronary artery, Coronary artery anomalies, Congenital heart disease, Computed tomography, Strain echocardiography

## Abstract

**Background:**

We report the case of a 63-year-old white Caucasian male patient admitted to the clinic because of atypical angina and palpitations. Other comorbidities included hypertension, hyperuricaemia, and hypercholesterolaemia. He was admitted to a tertiary cardiac centre for deepened diagnostics of his complaints.

**Case summary:**

Echocardiography revealed no pathology, but due to high calcium score (Agatston > 400 units), signs of arrhythmia during exercise, and atypical angina complaints, further investigations were performed. Invasive coronary angiography revealed stenosis up to 53% in the middle part of the right coronary artery (RCA), and computed tomography angiography showed no left coronary artery, only malignant-coursed vessel, running from the proximal part of the RCA. Single-photon emission tomography findings allowed to come to a decision not to perform invasive treatment (coronary artery bypass grafting), due to acceptable perfusion and viability of the heart muscle.

**Discussion:**

Single coronary artery is an extremely rare anomaly. This requires highly individualized diagnostic approaches, which include multiple imaging modalities, as each adds different information. While the only coronary vessel was narrowed in 53%, no significant ischaemia was detected. Left ventricular function remained preserved.

**Learning points**

- Heart single-photon emission computed tomography, as a nuclear medicine diagnostic method, allows to broaden the diagnostic process and observe not only morphological pathologies, but also the perfusion and viability of the heart muscle.
- Even if the patient is an adult, the possibility of anatomical, congenital coronary vessels pathologies should be considered, as well as their impact on the patient’s health condition.

## Introduction

Single coronary artery (SCA) is a rare congenital anomaly with a prevalence ranging from 0.0024% to 0.066% in the general population, often associated with other congenital heart defects.^1^ Typical clinical manifestations of coronary artery anomalies include angina, arrhythmias, and even sudden cardiac death; however, these anomalies can also be asymptomatic. The lifelong consequences of SCA can exacerbate the prognosis of cardiovascular diseases, particularly in geriatric patients who may experience progressive narrowing of the sole coronary artery. The literature describes a few cases of a single right coronary artery (RCA), predominantly diagnosed using invasive coronary angiography (ICA), or computed tomography angiography (CTA).^2^ This report details a rare case of SCA diagnosed using single-photon emission computed tomography (SPECT), specifically noting the combination of a single RCA anomaly with stenosis exceeding 50% and a long (58 mm) intramuscular course.

## Summary figure

**Figure:**
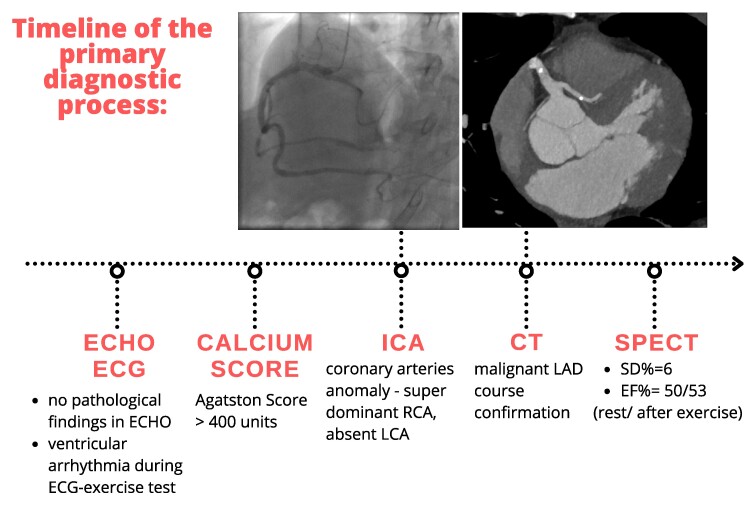


### Case report

We present the case of a 63-year-old white Caucasian male patient with atypical angina and palpitations who was admitted to our clinic. His medical history included hypertension, hyperuricaemia, and hypercholesterolaemia treated with beta-blocker, indapamide, allopurinol, angiotensin receptor blocker and statin. The echocardiographic evaluation revealed preserved systolic function of both ventricles and no valvular abnormalities. Due to his symptomatic presentation, ventricular arrhythmias (including single ventricular extrasystoles and non-sustained ventricular tachycardia), and Agatston score exceeding 400 units, ICA was indicated. According to guidelines, non-contrast computed tomography (CT) to assess calcium was performed before ICA to exclude patients with extremely high values from injecting contrast.

**Figure 1:**
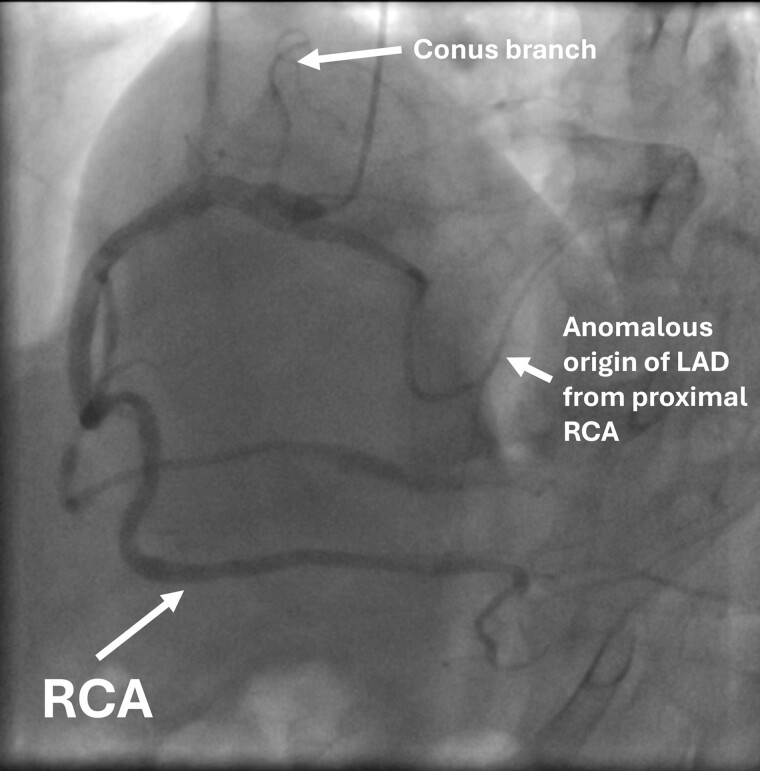
Invasive coronary angiography image—branches labelled.

**Figure 2:**
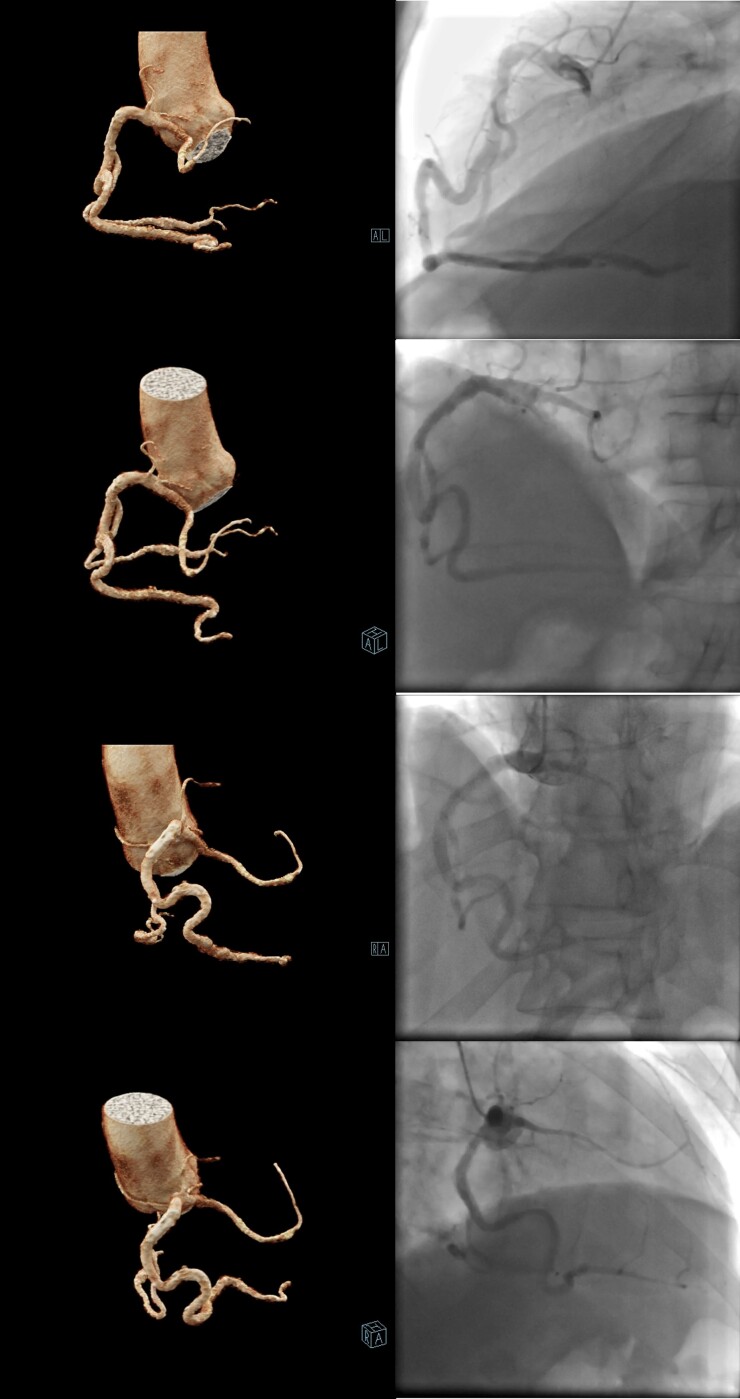
Invasive coronary angiography and computed tomography angiography images—the course of single right coronary artery.

Invasive coronary angiography and subsequent CTA confirmed a single RCA originating from the right coronary sinus (*Figure 1*). The RCA exhibited an unusual course: a small vessel branched from its proximal segment, traversed intramuscularly through the interventricular septum, and then followed a distal epicardial route before bifurcating into the proximal and distal left anterior descending arteries. The middle segment of the RCA, which supplied posterolateral and posterior descending artery branches, displayed calcification with stenosis up to 53%. Additionally, two epicardial arteries originating from the posterior descending artery and ascending along the left ventricular (LV) wall were identified as obtuse marginal branches (*Figure 2*).

Following a cardiac surgical consultation, a SPECT exercise test using 99mTc-MIBI was performed. The test revealed a partially reversible perfusion defect in the inferior wall and apex, with a summed difference score percentage (SD%) of 6%. Gated SPECT imaging demonstrated a LV ejection fraction of 57% and mild hypokinesis of the inferior wall. Based on these findings, pharmacological management was initiated in lieu of invasive cardiac surgery. A loop recorder was implanted for ongoing monitoring.

**Figure 3:**
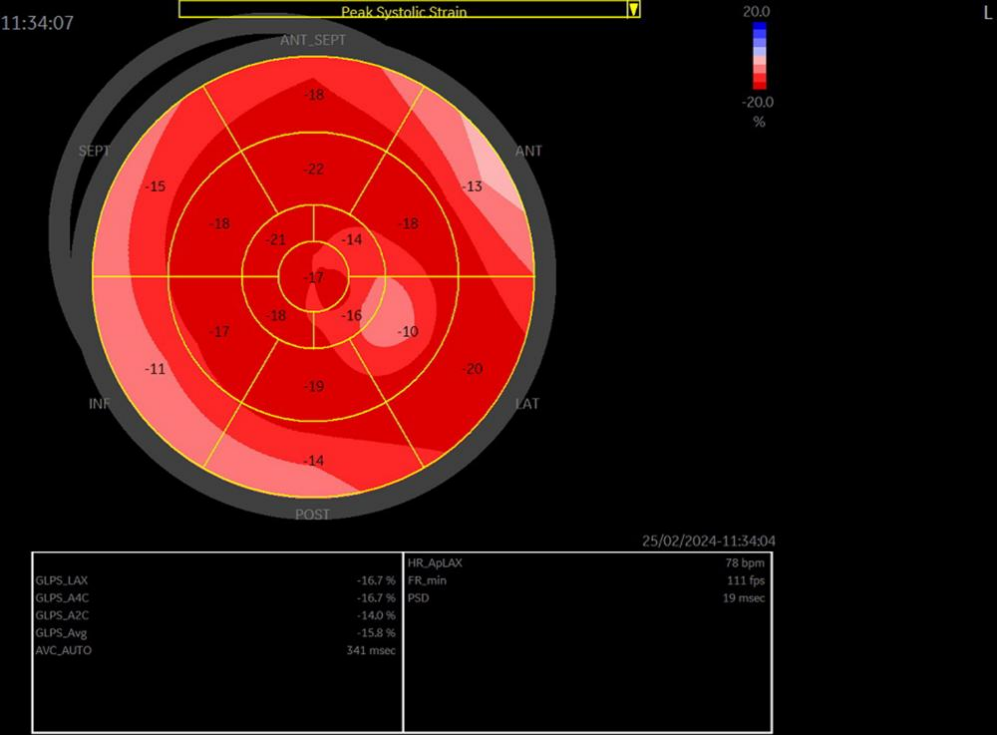
Strain echocardiography (‘bulls eye’)—the mildly decreased global longitudinal strain in the inferior wall, anterior wall, and apex.

**Figure 4:**
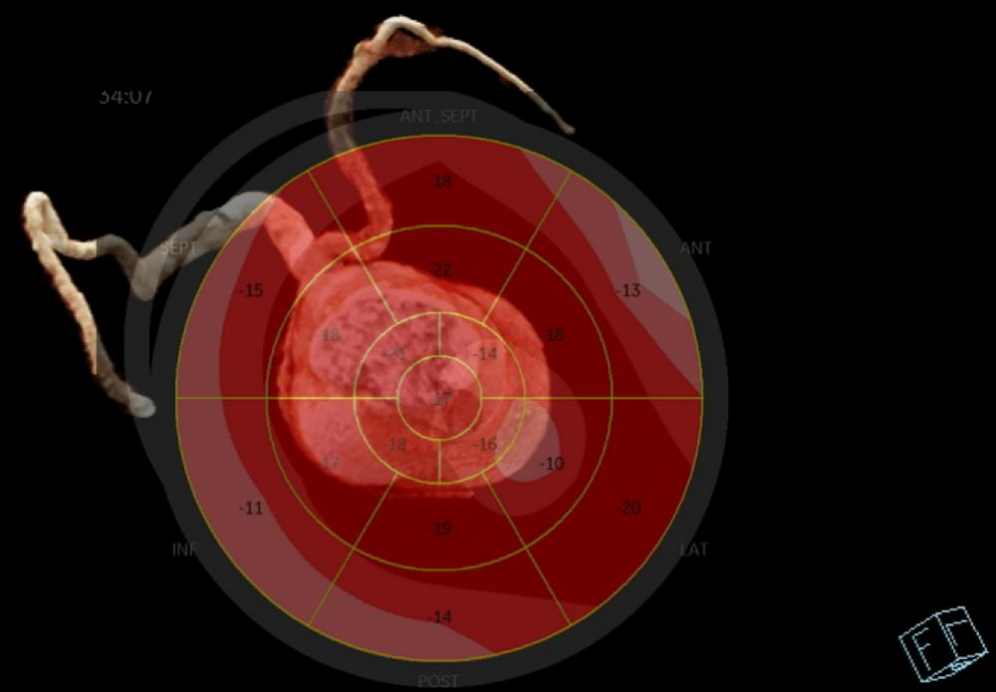
Fusion of strain echocardiography (‘bulls eye’) and computed tomography angiography images.

Single-photon emission computed tomography imaging was repeated annually, showing no evidence of clinical or perfusion deterioration over a 4-year follow-up period (SD%: 6%, 4%, 3%). Strain echocardiography conducted in the fourth year revealed mildly reduced global longitudinal strain (−11%) in the apex, inferior, and anterior walls (*Figures 3* and *4*). Currently, the patient does not report any complaints.

### Discussion

This case underscores the critical role of multimodality imaging in the diagnosis and management of rare coronary artery anomalies. Current guidelines do not give exact strategies for the rare cases, which makes each described case clinically valuable.^3^ Integrating nuclear imaging techniques such as SPECT with echocardiographic assessment facilitates the early detection of ischaemic regions and informs clinical decision-making. The collaborative efforts of a multidisciplinary team—including cardiologists, cardiac imagers, and cardiac surgeons—were essential in achieving a favourable long-term outcome in this patient. The non-invasive management strategy adopted in this case has proven effective, highlighting the importance of personalized treatment plans for patients with complex congenital coronary anomalies.

In conclusion, this report emphasizes the necessity for comprehensive diagnostic evaluation and multidisciplinary management in cases of rare coronary anomalies. The positive outcome observed over a 4-year follow-up period in this patient with a SCA underscores the efficacy of tailored medical therapy and the utility of advanced imaging modalities in guiding clinical decisions.

#### Conclusion

Coronary anomalies, especially rare ones like SCA, demand an individualized treatment plan in which multimodality imaging might be helpful.

## Supplementary Material

ytae584_Supplementary_Data

## Data Availability

The data underlying this article are available in the article and its online Supplementary material.

## Lead author biography

Alexander Suchodolski graduated from the Silesian Medical University in 2019 and now works at the Silesian Center for Heart Disease. His primary research interest is in cardiac imaging, specifically CT, magnetic resonance, and echocardiography. He is currently pursuing his PhD on the topic of coronary anomalies in adults under the guidance of M.S. and J.G. at the Silesian Medical University. He is an active member of various medical societies, demonstrating his dedication to staying up-to-date with the latest developments in his field.

## Supplementary material

ICA image—branches labelled.

Volume rendering of CTA—video.

## Notes

**Consent:** In accordance with COPE guidelines, patient informed consent was obtained for the publication of the case.

**Funding:** No funding needs to be reported in this research work.
